# Audiovisual integration and sensory dominance effects in older adults with subjective cognitive decline: Enhanced redundant effects and stronger fusion illusion susceptibility

**DOI:** 10.1002/brb3.3570

**Published:** 2024-08-27

**Authors:** Shengnan Li, Weiping Yang, Yueying Li, Ruizhi Li, Zhilin Zhang, Satoshi Takahashi, Yoshimichi Ejima, Jinglong Wu, Mengni Zhou, Jiajia Yang

**Affiliations:** ^1^ Cognitive Neuroscience Laboratory, Graduate School of Interdisciplinary Science and Engineering in Health Systems Okayama University Okayama Japan; ^2^ Department of Psychology, Faculty of Education Hubei University Wuhan China; ^3^ Graduate School of Humanities Kobe University Kobe Japan; ^4^ Research Center for Medical Artificial Intelligence, Shenzhen Institute of Advanced Technology Chinese Academy of Sciences Shenzhen China; ^5^ School of Medical Technology Beijing Institute of Technology Beijing China

**Keywords:** audiovisual integration, Colavita effect, facilitation effect, fission and fusion illusion, race modal

## Abstract

**Introduction:**

Subjective cognitive decline (SCD) refers to individuals’ perceived decline in memory and/or other cognitive abilities relative to their previous level of performance. Sensory decline is one of the main manifestations of decline in older adults with SCD. The efficient integration of visual and auditory information, known as audiovisual integration, is a crucial perceptual process. This study aims to evaluate audiovisual integration in older adults with SCD.

**Methods:**

We adopted the audiovisual detection task, the Colavita task, and the Sound‐Induced Flash Illusion (SIFI) task to evaluate the audiovisual integration by examining both redundant and illusory effects. Older adults diagnosed with SCD (N = 50, mean age = 67.8 years) and a control group of non‐SCD older adults (N = 51, mean age = 66.5 years) were recruited. All participants took part in the three aforementioned experiments.

**Results:**

The outcomes showed that a redundant effect occurred in both SCD and non‐SCD older adults, with SCD older adults gaining more benefits in audiovisual detection task. Moreover, an equivalent amount of the visual dominance effect was observed among both SCD and non‐SCD older adults in Colavita task. In addition, older adults with SCD perceived an equal fission illusion but a bigger fusion illusion compared with non‐SCD older adults in SIFI task.

**Conclusions:**

Overall, older adults with SCD exhibit increased audiovisual redundant effects and stronger fusion illusion susceptibility compared to non‐SCD older adults. Besides, visual dominance was observed in both groups via the Colavita task, with no significant difference between non‐SCD and SCD older adults. These findings implied that audiovisual integration might offer a potential way for the identification of SCD.

## Graphical abstract

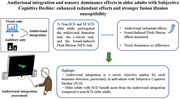

## 1 INTRODUCTION

Subjective cognitive decline (SCD) pertains to an individual's perceived decline in memory and/or other cognitive abilities compared to their previous level of functioning (Rabin et al., 2017; Reisberg et al., 1982). The potential risk for individuals with SCD to progress to MCI or Alzheimer's disease (AD) has attracted increasing attention. The identification of SCD in older adults primarily depends on subjective reports in many cases (Jessen et al., 2020). Additionally, sensory decline emerges as a manifestation of decline in older adults with SCD (Carr et al., 2019; Koppara et al., 2015). In terms of sensory perception, visual and auditory sensory modalities are important sources of external information, and the integration of visual and auditory information (either presented at the same time or at certain intervals) into a unified and coherent perceptual process is known as audiovisual integration (Freiherr et al., 2013; Laurienti et al., 2006). Compared to older adults without cognitive impairment, those with MCI and AD have been shown to exhibit a delayed onset of audiovisual integration (J. Wu, Yang, Gao, et al., 2012), an enlarged temporal binding window (TBW, which refers to the time window during which sensory signals from different modalities are likely to be integrated into a single perceptual event) (J. Chan et al., 2015), and a diminished McGurk effect (which refers to an auditory illusion where perception of a spoken sound is influenced by accompanying visual information, often resulting in the perception of a different sound) (Delbeuck et al., 2007; Festa et al., 2017). A recent study reviewed the alteration of audiovisual integrative behavioral symptoms in AD and emphasized the value of audiovisual integration alterations as potential signs for the early diagnosis and progression of AD (Liu et al., 2023). Considering the early sensitivity of abnormal responses in audiovisual integration among individuals with AD, it is imperative to investigate whether SCD is also susceptible to audiovisual integration.

Audiovisual integration primarily encompasses two types of integration effects: redundant effects and illusion effects. The redundant effect was defined as responses that are faster and more accurate when both visual and auditory information are presented simultaneously, compared to a single sensory modality (Peiffer et al., 2007). Age‐related audiovisual integration studies have demonstrated an enhanced audiovisual benefit for older adults (compared with that of younger adults) in audiovisual discrimination tasks (Diederich et al., 2008; Laurienti et al., 2006; Zou et al., 2017), sound‐induced flash illusion tasks (DeLoss et al., 2013; Hernández et al., 2019), and speech perception tasks (Sekiyama et al., 2014). Moreover, the time window of integration (which refers to the temporal interval during which cross‐modal stimuli presented simultaneously are perceptually integrated into a single perceptual event) is an important index to evaluate when audiovisual integration occurs (Diederich et al., 2008). Studies have found that the time window for older adults was longer but occurred later than that for young participants (J. Wu, Yang, Gao, et al., 2012). Compared to normal older adults, MCI and AD exhibited later onset times and peak benefits of redundant effects (J. Wu, Yang, Yu, et al., 2012). Given these findings, it is crucial to explore how specific cognitive conditions like SCD influence these integration patterns. This study aims to elucidate the changes in audiovisual redundant effects in older adults with SCD.

Another type of integration effect involves cross‐modality illusions, including the visual dominance Colavita effect and auditory dominant sound‐induced flash illusion (SIFI). The Colavita visual dominance effect refers to a phenomenon in multisensory perception where visual stimuli are given more attention or perceived as more dominant compared to auditory or other sensory stimuli. For example, in an experiment where participants are asked to detect a visual flash and an auditory beep presented simultaneously, participants might more readily notice or react to the visual flash while potentially missing or being less aware of the auditory beep (Spence, 2009). Previous studies have mainly focused on the developmental trajectory of the Colavita effect, focus on the sensory dominance changes during development in children (Nava & Pavani, 2013). Barnhart et al. (2018) and Hirst et al. (2018) examined the development of modality dominance in children, young adults, and older adults and found clear evidence of visual dominance in older adults. This study aimed to explore whether the visual dominance effect differs between older adults with SCD and non‐SCD. On the other hand, SIFI includes fission and fusion illusions occurring within a short temporal interval, which can help in investigating the temporal dynamics of multisensory integration. In the fission illusion, a single flash paired with two beeps is perceived as two flashes. Conversely, a fusion illusion occurs when two flashes are accompanied by a single beep but are perceived as one flash (Hirst et al., 2020). In normal aging, older adults typically require longer temporal offsets to detect asynchrony (Y. M. Chan et al., 2014); additionally, they have difficulty discriminating temporal order (Setti et al., 2011) and become more vulnerable to flash fission illusions induced by well‐separated sounds (Hernandez et al., 2019; McGovern et al., 2014). For patients with cognitive decline, J. Chan et al. (2015) found that MCI patients perceived significantly more fission illusions than healthy controls (HC) over longer auditory time intervals of up to 300 ms, which indicated that MCI patients may encounter challenges in suppressing irrelevant auditory stimuli, thus leading to an increased perception of illusory flashes (J. Chan et al., 2015). In addition, higher susceptibility to the fission illusion was predicted by older age and a lower score on the Montreal Cognitive Assessment (MoCA) (Hernandez et al., 2019). Overall, previous findings have provided evidence that MCI is sensitive to both visual and auditory dominance, and it remains to be explored whether older adults with SCD are also susceptible to cross‐modal illusions.

It has been suggested that research on audiovisual integration is essential, as it may facilitate the identification of more sensitive markers of age‐ and disease‐related cognitive decline (Freiherr et al., 2013). Research on the audiovisual integration characteristics of older adults with SCD, and the differences compared to those without SCD, may contribute to better identification of SCD. Hence, the current study aimed to examine whether individuals with SCD display distinct patterns of audiovisual integration when compared to those with normal older adults across a range of audiovisual perceptual tasks, including those exploring redundant effects, visual dominance Colavita effect, and auditory dominance SIFI effect.

## 2 EXPERIMENT 1

### 2.1 Participants

**FIGURE 1:**
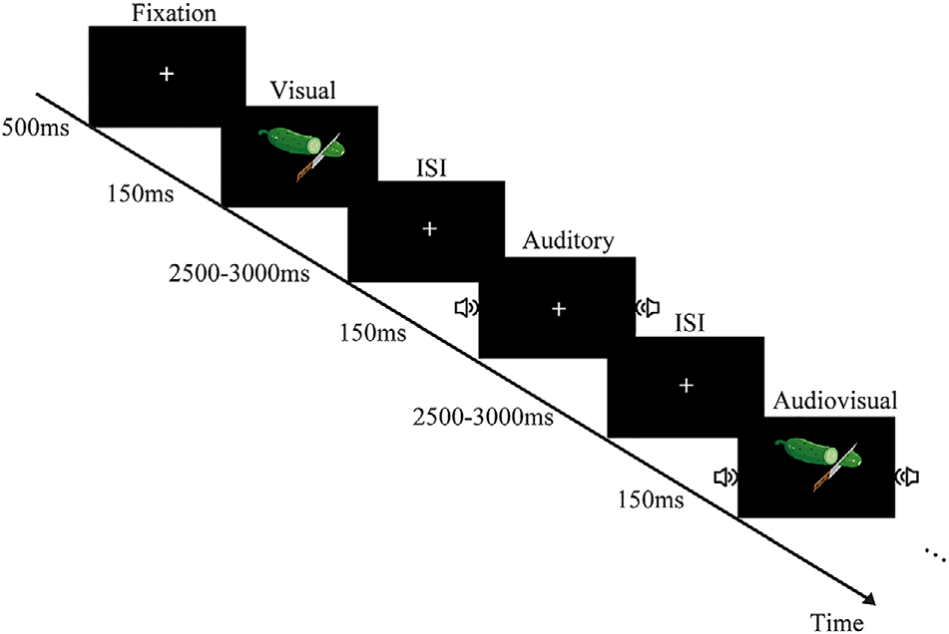
Schematic depiction of the experiment 1 design. ISI, inter‐stimulus intervals.

This is a randomized, controlled trial conducted in SCD and non‐SCD older adults. A total of 107 older adults were recruited through advertisements and completed three audiovisual experiments. Based on these references (Pan et al., 2020; Wang et al., 2019), inclusion criteria for all participants were as follows: aged more than 60 years; educated more than 6 years; having normal or corrected‐to‐normal vision and hearing to complete tests; having normal ability (≥27 points on the Mini‐Mental State Examination（MMSE）; ≥26 points on the Montreal Cognitive Assessment [MoCA]) (adjusted or age, sex, and education); preserved basic activities of daily living (ADL); not diagnosed with dementia or MCI by a neurologist; and clinical dementia rating (CDR, score < 0.5). Exclusion criteria were as follows: having a history of significant neurological and psychiatric illness (e.g., stroke, traumatic brain injury, depression, and others), or an unstable medical condition that might prevent them from performing cognitive test; Geriatric Depression Scale (11‐item) score ≤ 3; and Hamilton Depression Rating Scale (17‐item) score ≤ 12. After exclusions, the overall study cohort consisted of 101 older adults. All participants provided written informed consent to the experimental procedures, which were approved by the Shenzhen Institute of Advanced Technology, Chinese Academy of Sciences Research Committee and performed in accordance with the Declaration of Helsinki. An overview of the enrollment and study protocol is given in Figure 1.

### 2.2 Neuropsychological assessment

All of the participants completed the same neuropsychological testing battery to probe the current cognitive status of each older participant. The MMSE, MoCA, CDR questionnaires were used to assess possible age‐related MCI and AD. This study excluded individuals with MCI and AD and separated participants into SCD and non‐SCD groups via questionnaires and interviews. The definition of SCD was based on these features: participants reported a self‐experienced persistent decline in memory capacity, which is unrelated to an acute event; normal age‐, gender‐, and education‐adjusted performance on standardized cognitive tests that are above the cutoff for impairment in cognitive tests (Jessen et al., 2014). Subject cognitive decline questionnaire (9‐item, score > 4) was employed to find out about their memory loss and classify specific groups of participants. Importantly, these features, understood through the interviews, were also included in the SCD: onset of memory decline within the last 5 years; worry about their memory decline; and feeling of performing worse than others of the same age group (Jessen et al., 2020; Rabin et al., 2017).

In total, the data of 51 non‐SCD and 50 SCD older adults were included in these three experiments. Demographic characteristics and group differences are reported in Table 1.

**TABLE 1:** Group differences in demographic characteristics.

	Non‐SCD (N = 51)	SCD (N = 50)	Statistic	*p* value
Sex (M/F)	18/33	24/26	*χ* ^2^ = 0.085	.771
Age (years)	66.5 (5.3)	67.8 (4.5)	*t* = −1.327	.188
Age range (years)	61–78	60–75	–	–
Education (years)	11.3 (2.7)	10.0 (2.0)	*t* = 2.822	.006^*^
MMSE total	29.2 (1.1)	27.3 (1.7)	*t* = 6.418	.001^*^
MocA total	29.3 (1.1)	27.4 (2.0)	*t *= 6.132	.001^*^
SCD‐Q24	2.3 (2.0)	10.8 (3.9)	*t *= −14.051	.001^*^
SCD‐Q9	1.2 (1.2)	6.0 (1.7)	*t *= −16.775	.001^*^

*Note*: Data are presented as the mean and standard deviation (mean ± SD).

Abbreviation: SCD, subjective cognitive decline.

^∗^ denotes statistical significance at *p* < .05.

### 2.3 Stimuli and procedure

Stimulus presentation and recording were performed by using the software E‐Prime 3.0 (Psychology Software Tools, Inc.) in a sound‐ and light‐attenuated booth (laboratory room, Shenzhen Institutes of Advanced Technology, China). Stimulus presentation and recording of participants’ responses were implemented using the software E‐Prime 3.0 (Psychology Software Tools, Inc.). All visual stimuli were presented on a Dell with a screen resolution of 1024 × 768 pixels and a refresh rate of 60 Hz. Auditory stimuli were controlled by a USB audio interface (UR22mkII; Steinberg) and delivered through in‐ear headphones (ER30; Etymotic Research).

Experiment 1 is an audiovisual (AV) detection task. Visual target stimuli (V) for this task were a knife‐cut cucumber (5.2 cm × 12.75 cm, with a vertical visual angle of 5° and a horizontal visual angle of 12°) that was presented on a black background at the center of the screen. Auditory targets were the audio of the collision of the knife against a board. The auditory targets (A) were 32‐bit audio with a sampling rate of 48 kHz dealt with by the software Adobe Audition and were presented to the left and right ear simultaneously through earphones at approximately 72 dB sound pressure level (SPL) for 150 ms (including 10 ms of rise/fall cosine gate). The AV target was the simultaneous presentation of the visual and auditory stimuli. Inter‐stimulus intervals (ISI) varied from 2000 to 3000 ms for response and rest. The task consisted of 170 trials (V50, A50, AV50, and nontarget 20). For nontarget trials, participant responses were not needed. All of the stimuli were randomly presented. The participants were instructed to respond to target stimuli as rapidly and accurately as possible by pressing the left key of the mouse.

### 2.4 Data analysis

To control for the redundant nature of the audiovisual condition, RTs were analyzed using an independent race model. By using this model (P(A) + P(V) − P(A) × P(V)), response times to audiovisual stimuli were compared to the visual and auditory response times with the total probabilities (total probabilities refers to the combined probability of either the auditory or visual signal eliciting a response alone). If the probability of response to an AV stimulus is significantly greater than the probability of response predicted by the race model, it indicates that audiovisual integration occurred (Miller, 1986). Afterwards, the cumulative probability of difference was obtained by subtracting the race model curve from the generated audiovisual cumulative distribution function (CDF) curve for each subject in each 10‐ms bin and by performing a one‐sample *t* test (*p* ≤ .05 vs. 0) to derive the integration time window. The greatest audiovisual facilitation of the mean probability difference curves is defined as the peak benefit, and the time spanning from the presentation of the target to the maximal benefit is defined as the peak latency. Additionally, because each subject has a different time course of responses, averaging difference curves may not provide a complete indication of group differences. Positive area under the curve (pAUC) for significant time periods was calculated for each subject to avoid timing differences.

### 2.5 Results

**FIGURE 2:**
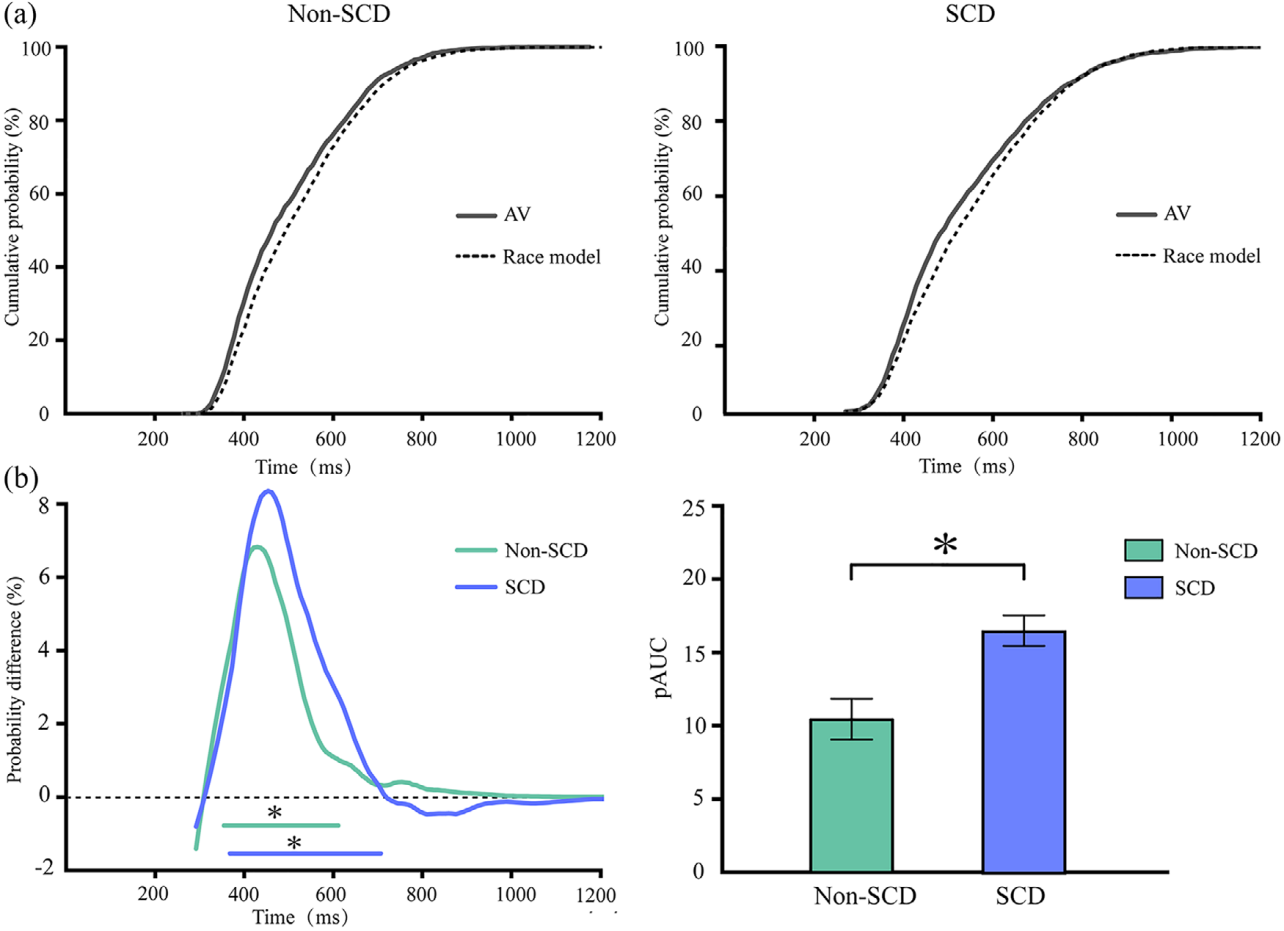
(a) Cumulative distribution functions (CDFs) for non‐subjective cognitive decline (non‐SCD) (left) and SCD (right) older adults. (b) Left: probability difference between audiovisual CDFs and race model CDFs for non‐SCD and SCD older adults. *The time when the peak (maximum probability value) occurs; right: Positive area under the curve (pAUC) area size for non‐SCD and SCD older adults. *p <.05, ** p <.01. AV, audiovisual.

Table 2 shows the accuracy and mean RTs for the two groups. Figure 2a shows the cumulative probability difference curves in non‐SCD and SCD older adults. Two‐tailed t tests were conducted between the audiovisual CDFs and the race model to evaluate the redundant effect in each 10‐ms time bin under each condition. For non‐SCD, the integration window is 320–580 ms, with a peak of 6.12% at 400 ms. Nevertheless, the SCD older adults had a broader time window from 330 to 650 ms, and the peak was 8.24% at 460 ms. An analysis of *t*‐independent samples test for peak latency was conducted to compare the onset time of audiovisual integration. A delayed audiovisual integration effect of SCD compared to that of non‐SCD older adults was observed, *t* (99) = −2.326, *p* = .022, with a peak latency of SCD (480 ms) that was longer than in the non‐SCD older adults (442 ms). In addition, an independent t test was performed on the positive AUC for both groups. The results indicated that the pAUC for SCD (*M* = 16.36, SE = 18.48) was greater than that for non‐SCD older adults (*M* = 10.11, SE = 18.48), *t* (99) = 3.861, *p* < .05, as indicated in Figure 2b.

**TABLE 2:** The mean hit rates and response times for both non‐subjective cognitive decline (non‐SCD) and SCD older adults.

	Hit rate (%)		Response times	
conditions	Non‐SCD	SCD	Non‐SCD	SCD
Visual	98.08 (4.3)	95.76 (5.5)	604 (10.1)	672 (16.6)
Auditory	98.24 (2.8)	98.68 (2.2)	561 (14.1)	579 (16.3)
Audiovisual	99.21 (2.1)	98.68 (2.3)	505 (11.0)	521 (13.5)

*Note*: Data are presented as the mean and standard deviation (mean ± SD).

### 2.6 Discussion

Two main results emerged from Experiment 1. First, the audiovisual redundant effect (i.e., a faster audiovisual stimulus than auditory‐only and visual‐only responses, as well as violation of race model) was present in non‐SCD and SCD older adults. Notably, a greater pAUC and peak benefit of SCD older adults indicated that SCD older adults benefitted more from the audiovisual stimuli. These results align with prior research that has identified a pronounced redundant effect in both MCI and AD patients (J. Wu, Yang, Yu, et al., 2012). Second, the onset time of integrating audiovisual stimuli occurred later, which indicates that the audiovisual redundant effect in SCD was delayed. Similar results were reported in MCI and AD (J. Wu, Yang, Gao, et al., 2012).

It is well known that older adults experience deficits in visual and auditory abilities, such as a decrease in visual and auditory acuity (Chou et al., 2016; Jayakody et al., 2018). Previous studies have reported a visual dominance in older adults, which implies that, compared to auditory input, older adults tend to rely more on visual information for processing and interpreting the surrounding world (Barnhart et al., 2018; Diaconescu et al., 2013). To explore whether SCD has an impact on visual dominance, the Colavita effect was explored in Experiment 2.

## 3 EXPERIMENT 2

### 3.1 Stimuli and procedure

**FIGURE 3:**
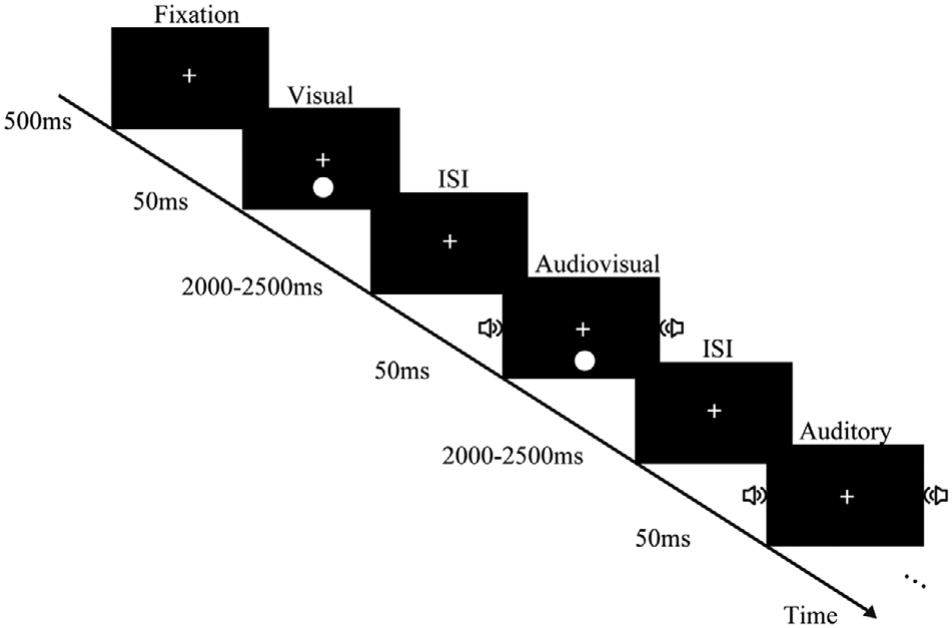
Schematic depiction of the experiment 2 design. ISI, inter‐stimulus intervals.

The apparatus was the same as in Experiment 1. The visual stimulus consisted of a white circle with luminance of 120 cd/m^2^ subtending 1.4° at 7° eccentricity. The fixation was a white “+” (0.5° × 0.5°) that appeared in the center of the screen. The auditory target was a 1000 Hz pure tone burst, presented at 65 dB in dual channels of headphones. There were three types of trials: unimodal auditory trials, unimodal visual trials, and bimodal trials in which the auditory and visual targets were presented simultaneously. Both visual and auditory targets were presented for 50 ms. There were 600 trials in total, with the ratio of visual, auditory, and audiovisual stimuli set to 2:2:1. Participants were instructed to press the left key if they saw the visual target and to press the right key if they heard the auditory target; additionally, they would press both keys if they saw the bimodal simultaneously presented stimulus. The key response involved a counterbalance among the participants. All of the participants were instructed to respond as quickly and accurately as possible (Figure 3).

### 3.2 Data analysis

In addition to the two types of unimodal trials, the bimodal trials were categorized into the following six types according to the performance of participants: (1) the Visual_Auditory (VA) responses, in which participants first responded to the visual component and then to the auditory component; (2) the Auditory_Visual (AV) responses, in which participants first responded to the auditory component and then to the visual component; (3) the “simultaneous” responses, in which participants responded simultaneously to the auditory and the visual components by pressing down the two response buttons at the same time, and when the absolute difference in response times to visual and auditory stimuli is less than 5 ms (|Visual_RT—Auditory_RT| < 5 ms) were categorized as the simultaneous trials as well; (4) the Visual_only responses, in which participants responded only to the visual component but not to the auditory component; (5) the Auditory_only responses, in which participants responded only to the auditory component but not to the visual component; (6) the “Missed” trials, in which participants had no responses. In Experiment 2, we explored the impact of the Colavita effect by examining two key measures: the proportion of responses and response times (RTs). The proportion of trials for each condition was determined by calculating the ratio of the number of bimodal trials specific to that condition to the total number of bimodal trials conducted.

The unimodal Auditory_only and Visual_only trials (incorrect bimodal trials) and the bimodal Auditory_Visual and Visual_Auditory trials (correct and nonsimultaneous bimodal trials) were the four conditions of interest. We initially analyzed the incorrect trials from the bimodal trials to examine the differences in the Colavita effect between non‐SCD and SCD older adults and submitted a 2 (group: non‐SCD and SCD) × 2 (type of trials: Visual_only and Auditory_only) repeated‐measures analysis of variance (ANOVA) on the proportion. Additionally, we carried out similar analyses for the proportion in which the participants pressed two keys at different times. This is the correct response to bimodal trials that consisted of Visual_Auditory and Auditory_Visual trials conditions. Finally, according to the previous study (E. Nava & F. Pavani, 2013), to illustrate the differences between the group difference, a 2 (group: non‐SCD and SCD) × 2 (target modality: auditory and visual) × 2 (target type: unimodal and bimodal) mixed‐factors ANOVA with the group as a between‐subjects factor and target modality (auditory and visual), target type (unimodal and bimodal) as within‐subject factors was submitted for RTs.

### 3.3 Results

**FIGURE 4:**
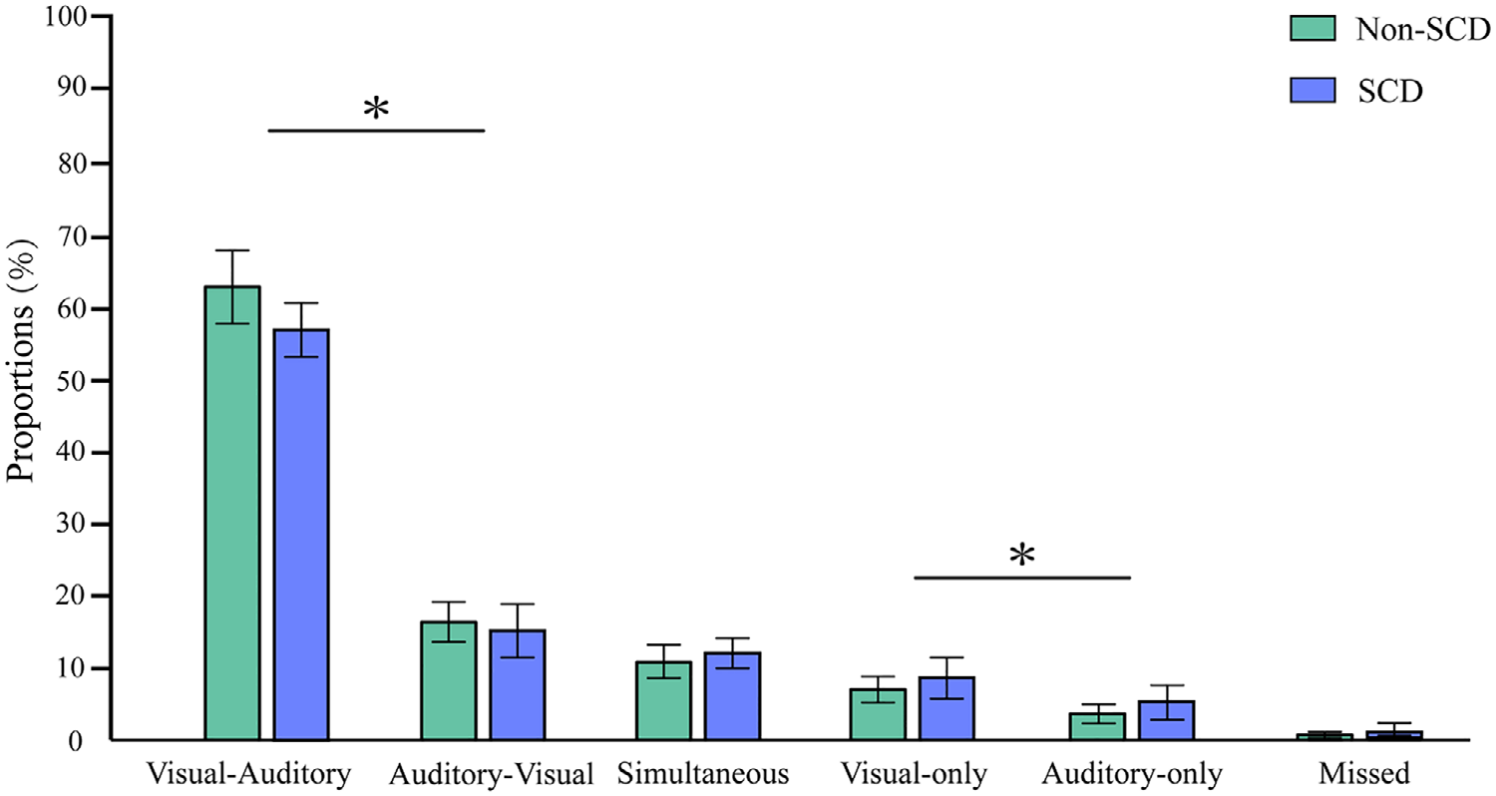
Proportions of the six different types of conditions in the bimodal trials of the non‐subjective cognitive decline (non‐SCD) and SCD older adults in experiment 2. *p <.05, ** p <.01.

Figure 4 describes the proportions of responses for the six different types of conditions in the bimodal trials for both groups. The ANOVA revealed a significant main effect for the type of trials (*F* (1, 98) = 10.790, *p* = .001), indicating that the proportion of incorrect responses in Visual_only trials (7.75%, SE = 0.8%) was significantly higher than in Auditory_only trials (4.76%, SE = 0.5%). This finding supports the presence of the Colavita effect. However, no significant main effect of the group, *F* (1, 98) = 3.106, *p* = .081, or interaction between group and type of trials was observed, *F* (1, 98) = 0.004, *p* = .949. Subsequent ANOVA for the proportions of Visual_Auditory and Auditory_Visual trials revealed a significant main effect of group (*F* (1, 98) = 6.965, *p* = .010), with the response proportions for non‐SCD older adults (79.29%, SE = 4.6%) being significantly greater than those for SCD older adults (74.06%, SE = 5.2%). The main effect of type of trials was observed, *F* (1, 98) = 387.507, *p* < .001, thus indicating a higher response proportion to the Visual_Auditory trials than to the Auditory_Visual trials. There was no significant interaction between group and type of trials. No difference in the Colavita effect was observed between the two groups, *t* (48) = 0.768, *p* = .446, Cohen's *d* = 0.11.

**FIGURE 5:**
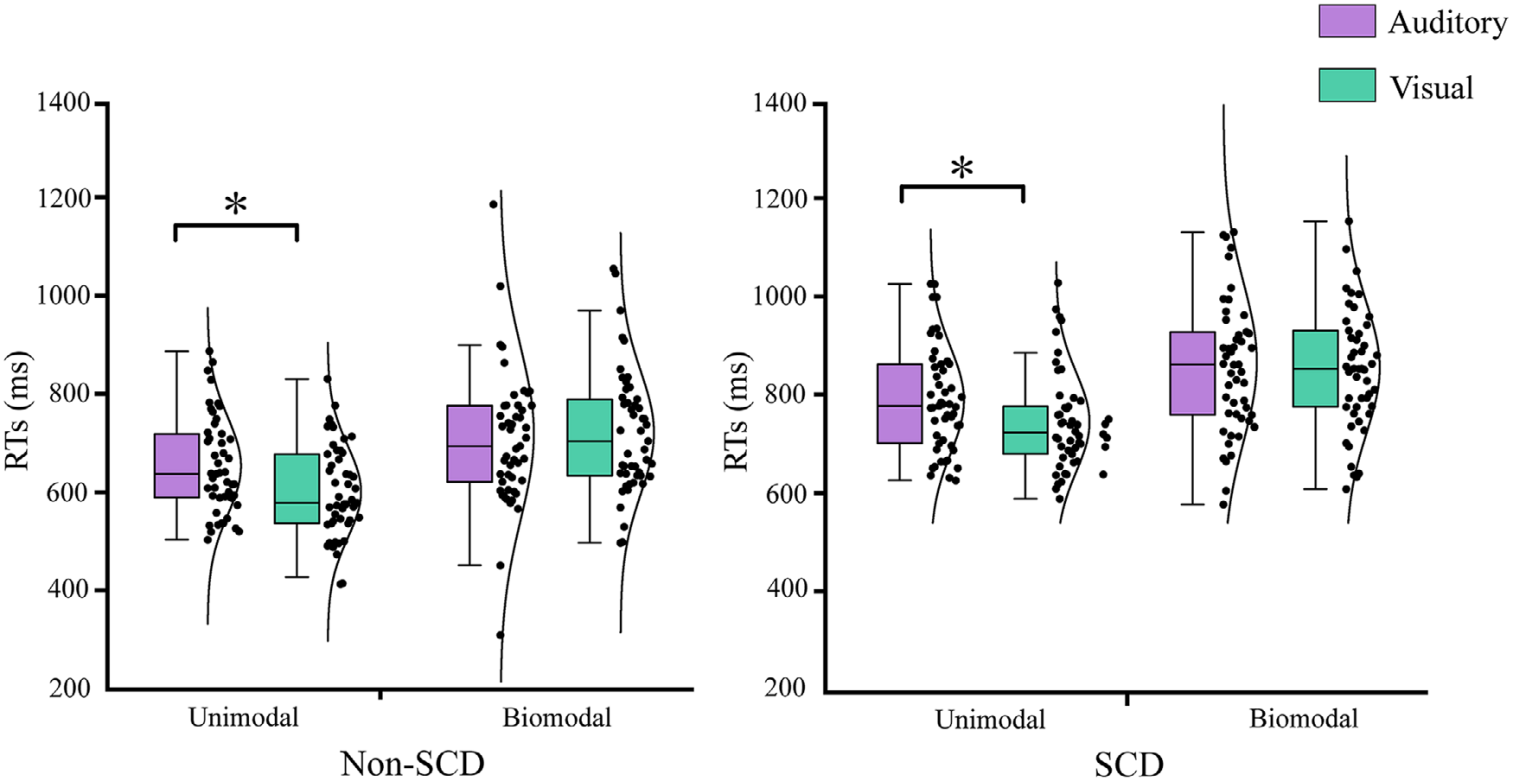
The differences in response times (RTs) between non‐subjective cognitive decline (non‐SCD) and SCD older adults under different type. *p <.05, ** p <.01.

For the response times (RTs), the analysis results demonstrated a significant main effect of group, *F* (1, 97) = 5.999, *p* = .016, and target type, *F* (1, 97) = 114.905, *p* < .001. The results indicated a faster response in non‐SCD older adults (709 ms, SE = 17 ms) than in SCD older adults (769 ms, SE = 17 ms), as well as faster responses in unimodal (689 ms, SE = 11 ms) than in bimodal (790 ms, SE = 15 ms) conditions. This analysis also demonstrated a significant main effect of target modality, *F* (1, 97) = 12.738, *p* = .001, with faster responses to visual stimuli (727 ms, SE = 13 ms) than auditory stimuli (751 ms, SE = 12 ms). A three‐way interaction between target type, target modality, and group was also marginally significant (*F* (1, 97) = 0.344, *p* = .059), so we further separately conducted a subsequent ANOVA analysis for both non‐SCD and SCD older adults. Further simple effect analysis revealed that norma non‐SCD and SCD old adults were faster in responding to visual than auditory unimodal stimulation. Specifically, non‐SCD older adults showed a RTs difference of 53 ms, responding faster to visual than to auditory stimuli in unimodal. Similarly, SCD older adults demonstrated a quicker RT to unimodal visual stimuli by 52 ms compared to unimodal auditory stimuli. However, in the bimodal condition, no significant difference was observed between the responses to visual and auditory stimuli (*p* > .05) (see Figure 5).

### 3.4 Discussion

**FIGURE 6:**
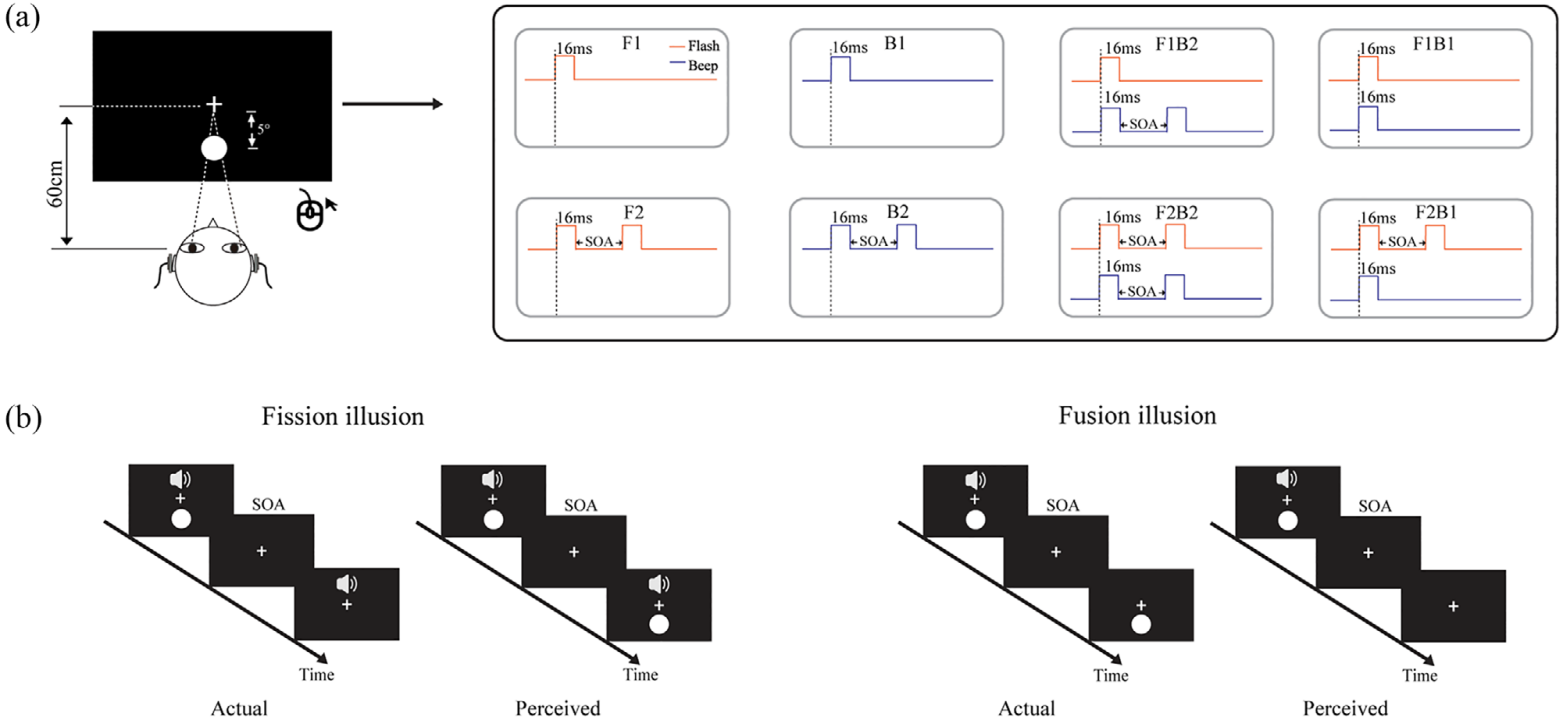
Schematic depiction of the experiment 3 design. SOA, stimulus onset asynchrony. *p <.05, ** p <.01.

Our results demonstrated that the visual dominance Colavita effect is a robust experimental phenomenon that exists in both SCD and non‐SCD older adults. Specifically, for both SCD and non‐SCD older adults, the proportion of Visual_only responses were significantly higher than that of Auditory_only responses, and the proportion of Visual_Auditory responses was significantly higher than that of Auditory_Visual responses. For RTs, the responses to the visual component of the unimodal stimuli were significantly faster than responses to the auditory component. These results are consistent with those of previous studies, thus demonstrating that the visual dominance effect is a prevalent phenomenon (Koppen & Spence, 2007a; Koppen & Spence, 2007b; Spence, 2009). Previous studies found that modality dominance effects appear to change across development (Hirst et al., 2018). Auditory dominance persists beyond 4 years of age but starts to change toward visual dominance in the early school years (Nava & Pavani, 2013). Other studies suggest that visual dominance effects may continue to strengthen into late adulthood (Diaconescu et al., 2013; Sekiyama et al., 2014). Consistent with prior research (Barnhart et al., 2018; Parker & Robinson, 2018), we observed the Colavita effect in both groups of older adults; however, no significant differences were detected between the groups, which suggests that the underlying mechanisms that promote visual dominance may be similar (Figure 6).

Besides, different sensory modalities may share and compete for resources (Sinnett et al., 2007). This competition suggests that when multiple sensory inputs are processed, the brain allocates available cognitive and neural resources, potentially prioritizing one modality over others (Huang et al., 2015). The allocation might be influenced by factors such as perceptual salience (DeLoss et al., 2013) and age‐related sensory deficits (Barnhart et al., 2018). Given these dynamics, it remains an open question whether the observed shift from auditory to visual dominance will persist or change with aging. It is particularly uncertain whether older adults will continue to exhibit stronger visual dominance effects compared to adults with SCD, who may experience different patterns of sensory processing due to their condition. Further studies are needed to explore how these sensory dominance patterns evolve with age and cognitive decline. Therefore, in Experiment 3, we further investigated the impact of SCD on the auditory domain from the perspective of the SIFI.

## 4 EXPERIMENT 3

### 4.1 Methods

The apparatus was the same as in Experiment 1. The visual stimulus was an annulus presented at maximum luminance and displayed for 8.33 ms (a single video frame). The inner and outer edges of the annulus stimulus extended 8.5° and 10° from the center of the screen. The auditory stimulus was a 1000 Hz pure tone presented for a duration of 16 ms at a 65 dB sound pressure level. Conditions known to produce fission (1F2B) and fusion (2F1B) illusions were randomly interleaved with unisensory (1F, 2F, 1B, and 2B) and multisensory (1F1B and 2F2B) control trials. In conditions containing two flashes or two beeps, auditory and visual stimuli were separated by different stimulus onset asynchronies (SOAs) of ±100, ±75, ±50, and ±25 ms, wherein positive and negative values indicated visual‐lead and auditory‐lead trials, respectively. Participants were instructed to press the left mouse button if they perceived one visual flash and to press the right mouse button if they perceived two visual flashes while ignoring the task's irrelevant auditory beeps. Key response keys were counterbalanced across the participants.

### 4.2 Data analysis

First, a mixed ANOVA with a 2 (group: non‐SCD and SCD) × 8 (SOA) × 2 (conditions: fission and fusion) design was conducted to investigate differences in the proportion of fission and fusion illusions and whether the main effect of the group occurred. To further investigate whether the group difference occurred on fission illusion or fusion illusion, a 2 (group: non‐SCD, SCD) × 8 (SOA) mixed ANOVA should be conducted for the fission and fusion conditions, respectively. Additionally, to further clarify the interaction between the cognitive of older adults and the shape of the TBW for 1F2B and 2F1B trials, a curve‐fitting procedure was employed to establish the TBW for both group‐averaged data. Data were fit with two sigmoid curves generated using the MATLAB R2021b Gaussian distribution function, splitting the data into left (auditory presented first) and right (visual presented first) sides and fitting them separately (0 ms included on both sides). Curve fits that produced an R2 of less than 50% were excluded from this analysis and then fitted with sigmoid (Equation 1) as the activation function.

(1)

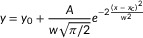




### 4.3 Results

The first ANOVA was performed and demonstrated that there was a significant main effect of conditions, *F* (1, 98) = 164.942, *p* < .001, thus indicating a higher proportion of fission illusory responses (71.8%, SE = 3.1%) than fusion illusory responses (28.9%, SE = 3.0%). The results also showed that the main effect of groups was significant, *F* (1, 98) = 7.646, *p* = .007, thus indicating a greater illusory response in SCD older adults (57.4%, SE = 3.6%) than in non‐SCD older adults (43.4%, SE = 3.6%). The main effect of SOA, *F* (7, 686) = 6.472, *p* < .001, and the interaction effect between SOA and conditions were significant, *F* (7, 686) = 2.417, *p* = .024.

**FIGURE 7:**
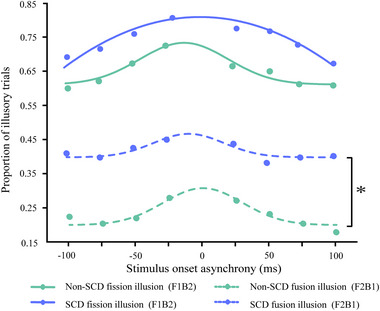
Mean proportion of illusion responses in the fission (F1B2) and fusion (F2B1) conditions as a function of stimulus onset asynchrony for non‐subjective cognitive decline (non‐SCD) and SCD older adults.

The analysis of the F1B2 trial revealed the impact of fission illusion between non‐SCD and SCD older adults. The second ANOVA analysis demonstrated a significant main effect of SOA, *F* (7, 686) = 4.994, *p* < .001, thus indicating that susceptibility to illusion was affected by different SOAs. However, there was no significant main effect of group (*F* (1, 98) = 2.183, *p* = .143) or interaction between group and SOA (*F* (7, 686) = 0.512, *p* = .798, *ηp* ^2^ = 0.005), which suggests that the magnitude and temporal limits of the fission illusion were very similar across the non‐SCD and SCD older adult groups. Similar to the fission illusion condition, a mixed ANOVA was applied to the fusion illusion (F2B1). The results showed a significant main effect of SOA (*F* (7, 686) = 3.898, *p* < .001), which is similar to the results of a previous study that demonstrated that time of stimulus asynchronies can affect the illusion perception. ANOVA also demonstrated that there was a main effect of group (*F* (1, 98) = 10.072, *p* = .002), thus indicating a larger fusion illusion in SCD older adults (38.3%, SE = 4.2%) than in non‐SCD older adults (19.5%, SE = 4.2%) (see Figure 7). However, no significant interaction between the group and SOA, *F* (7, 686) = 0.776, *p* = .592, was found.

### 4.4 Discussion

In Experiment 3, the results indicated that both non‐SCD older adults and SCD were susceptible to SIFI. Similar results have been found in healthy older adults (Hernandez et al., 2019; McGovern et al., 2014). Susceptibility to SIFI may serve as an indicator of the ability to process audiovisual information, with greater susceptibility to illusions indicating a stronger audiovisual effect (DeLoss et al., 2013). Our results indicated that SCD older adults showed significantly higher fusion illusion rates than non‐SCD older adults, whereas no group differences in SIFI fission were observed in both groups. Prior research has established that the fission illusion seems overall more reliable and stronger than the fusion illusion and more invulnerable to inter‐individual variability (Andersen et al., 2004; Innes‐Brown & Crewther, 2009), which might explain why we did not observe a difference in fission illusion between the groups.

The varying impact of SCD on fission and fusion illusions may be attributed to the complex interactions between sensory processing and cognitive factors. This notion is supported by studies investigating the SIFI in special populations, such as individuals with autism spectrum disorders (ASD) (Bao et al., 2017) and patients with unilateral spatial neglect (USN) (Bolognini et al., 2016) and schizophrenia (Vanes et al., 2016). For example, adolescents and adults with ASD and typically‐developing (TD) individuals were shown to have similar susceptibility to a fission illusion. However, the ASD group was significantly more susceptible to the fusion illusion (Bao et al., 2017). Additionally, a reliable “fusion” illusion is evoked in USN patients but not in healthy participants. Based on previous studies, the observed differences in susceptibility to fission and fusion illusions between SCD and non‐SCD older adults suggest that they may be integrating sensory information in a different manner. Moreover, further research is needed to understand the neural mechanisms by which the fission and fusion illusions differ between the groups and how cognitive decline may impact the neural processes underlying these illusions.

## 5 GENERAL DISCUSSION

In this study, we explored audiovisual integration and dominance effects among older adults with and without SCD. Experiment 1 demonstrated that both groups experienced audiovisual facilitation, with SCD participants showing a notably enhanced audiovisual redundant effect. Experiment 2 revealed visual dominance among two groups older adults through the Colavita task. However, there was no significant difference between normal and SCD older adults. Experiment 3 assessed susceptibility to visual and auditory illusions in the sound‐induced flash illusion (FISI) task and revealed a similar susceptibility to fission illusions across groups, but SCD individuals exhibited a higher tendency toward fusion illusions. These results suggests a differential integration of sensory information in SCD compared to normal aging.

### 5.1 Enhanced audiovisual integration in SCD older adults

Our study found enhanced redundant effects and stronger fusion illusion susceptibility in SCD older adults. These findings suggest that SCD individuals may actively engage in audiovisual integration as a compensatory mechanism, potentially to counter early neurodegenerative changes (Freiherr et al., 2013). This enhanced integration may help to compensate for the unimodal sensory deficits in SCD older adults, such as those documented in visual feature binding (Koppara et al., 2015) and contrast sensitivity performance (Risacher et al., 2013) as well as auditory processing challenges (Carr et al., 2019; Jayakody et al., 2020). Furthermore, neurophysiological studies provide additional evidence, showing that older adults with SCD might increase functional activity in brain regions to maintain the normal performance, which supports the existence of compensatory mechanisms in SCD older adults (Sun et al., 2016; Yang et al., 2018). Previous studies of resting‐state brain activity in individuals with SCD have shown notable differences when compared to healthy controls (HCs). Specifically, there was a significant increase in activity in several brain regions of SCD older adults, including the inferior parietal lobule (IPL), right superior temporal gyrus, right inferior temporal gyrus, fusiform gyrus (FG), and the right posterior lobe of the cerebellum (Han et al., 2012). A recent study also measured the fractional amplitude of low‐frequency fluctuation (fALFF), regional homogeneity (ReHo), and functional connectivity (FC) and revealed functional and structural alterations in SCD. Results found that the SCD group exhibited higher fALFF values than in the left inferior occipital gyrus (IOG) and left preCG (precentral gyrus) and showed increased FC in the left IPL compared to HCs (H. Wu et al., 2023). Besides, using magnetoencephalography (MEG), researchers have observed a significant increase in activation in the dorsal pathway, which includes the premotor and the dorsolateral prefrontal regions. The ventral pathway, formed by the ventral prefrontal region, the temporal lobe, and the IPL, also showed increased activity in late latency windows in SCD older adults as compared to HCs (Maestu et al., 2011). These studies displaying greater activation during different studies in SCD older adults may reflect the deployment of compensatory processes (Si et al., 2020). It is critical to note that our findings specifically highlight alterations unique to the audiovisual integration of SCD older adults. Future studies could use neuroimaging or electrophysiological techniques to explore how specific changes in cognitive processing in SCD influence sensory integration and provide a deeper insight into these compensatory mechanisms.

Another potential explanation for enhanced audiovisual integration in SCD older adults may be attributed to the decline in attention. Numerous behavioral and electroencephalographic studies have provided evidence for the existence of attentional deficits in SCD older adults (Peter et al., 2019; Tu et al., 2018). Attention‐associated P3 amplitudes attenuated in those individuals with SCD relative to HCs (Smart et al., 2014). Prior research has also shown that older adults may struggle to focus on a single stimulus and tend to bind all of the available information due to the deficit in attentional control (Jones & Noppeney, 2021). These findings further indicate that the same auditory and visual stimuli could naturally elicit more attention from SCD compared with non‐SCD older adults. Therefore, it is reasonable to observe an enhanced audiovisual integration in older adults with SCD.

### 5.2 Similar visual dominance effects across both groups

The findings from Experiment 2 show that participants consistently responded more quickly and more often to visual stimuli compared to auditory ones, indicating a visual dominance effect occurred. This pattern was observed across both older age groups, aligning with findings from previous research (Barnhart et al., 2018; Diaconescu et al., 2013). As aging progresses, declines in auditory sensitivity, particularly at high frequencies, and the susceptibility of auditory information to environmental noise may lead older individuals to depend more on visual information (Parker & Robinson, 2018). Furthermore, aging is associated with declines in attention and processing speed (Wahl et al., 2010), making visual information a more reliable and accessible source for object recognition, especially in situations of compromised hearing (Sekiyama et al., 2014). Previous studies revealed that older adults show a more pronounced visual dominance effect (Barnhart et al., 2018; Diaconescu et al., 2013). Notably, our study found no significant differences in visual dominance between the SCD and non‐SCD groups, suggesting that the underlying mechanisms that promote visual dominance may be similar across both groups, irrespective of the cognitive impairments typically seen in SCD. This observation calls for further research to delve into why SCD does not exacerbate sensory dominance patterns and to determine if other factors could be influencing these results.

## 6 CONCLUSIONS

To summarize, this study provides further evidence that older adults with SCD showed greater audiovisual integration and retained the visual dominance of their perception. This research may offer cost‐effective screening tools for identifying SCD and contribute to a more comprehensive understanding of its detection. From a clinical point of view, tracing how audiovisual integration develops in SCD may contribute to the understanding of the process of neurodegenerative diseases.

## AUTHOR CONTRIBUTIONS

Shengnan Li and Weiping Yang developed the idea for this study and designed it. Shengnan Li and Yueying Li collected, analyzed the data and drafted this manuscript. Jinglong Wu, Satoshi Takahashi, Yoshimichi Ejima, and Zhilin Zhang commented and edited the draft for journal publication. Jiajia Yang, Ruizhi Li, and Mengni Zhou participated in subject recruitment and provided consultation. All authors read and approved the final manuscript.

## CONFLICT OF INTEREST STATEMENT

The authors declare no conflicts of interest.

### PEER REVIEW

The peer review history for this article is available at https://publons.com/publon/10.1002/brb3.3570

## Data Availability

The datasets used and/or analyzed during the current study are available from the corresponding author on reasonable request.
